# Performance of whole-blood interferon-gamma release assay in patients admitted to the emergency department with pulmonary infiltrates

**DOI:** 10.1186/1471-2334-11-107

**Published:** 2011-04-24

**Authors:** Yoon Jee Lee, Jaehee Lee, Yi Young Kim, Dong Il Won, Seung Ick Cha, Jae Yong Park, Tae Hoon Jung, Chang Ho Kim

**Affiliations:** 1 Department of Internal Medicine, Kyungpook National University, School of Medicine, Daegu, Republic of Korea; 2 Clinical Pathology, Kyungpook National University, School of Medicine, Daegu, Republic of Korea

## Abstract

**Background:**

This study was conducted to evaluate the performance of a whole-blood interferon-gamma release assay in inpatients who were admitted to the emergency department (ED) with pulmonary infiltrates who required a differential diagnosis with pulmonary tuberculosis (TB).

**Methods:**

The patients with pulmonary infiltrates who received a QuantiFERON (QFT) test in the ED were included as an inpatient group and were divided into TB and non-TB group based on the final diagnosis. Patients with pulmonary TB who were tested in the outpatient department served as a control group.

**Results:**

In total, 377 QFT tests were analyzed. Of the 284 inpatient QFT tests, 29.6% had an indeterminate result (35.2% in the 196 patients with non-TB and 17.0% in the 88 patients with TB). In contrast, only 1.1% of the 93 outpatients with TB returned an indeterminate result (p < 0.001). The indeterminate QFT results in the inpatient group were independently associated with lymphocytopenia, hypoalbuminemia, and high C-reactive protein levels. Non-positive QFT results in inpatients with TB were associated with lymphocytopenia and hypoalbuminemia, while non-positive QFT results in outpatients with TB were associated with high erythrocyte sedimentation rates and radiographically more severe diseases.

**Conclusions:**

QFT tests in ED-based inpatients with pulmonary infiltrate return indeterminate results relatively frequently. In addition, inpatients and outpatients with pulmonary TB may differ in terms of the risk factors on non-positive QFT results.

## Background

The whole-blood interferon-gamma release assay (IGRA) is a new type of rapid, immune-based blood test that has significantly aided the diagnosis of tuberculosis (TB) infection [1-3]. However, since the test measures an immunological response, its performance is likely to be affected by differences in mycobacterial activity and factors that suppress the host's immune system, in particular cellular immunity. Indeed, we often encounter patients whose IGRA has returned an indeterminate result. The high rate of indeterminate results has led to concern about using new IGRAs, especially when dealing with patients whose cellular immunity is likely to be impaired [4,5]. However, to date, few studies have sought to identify the factors that are associated with the indeterminate results of IGRA test [6-10].

To address these issues, this study was conducted to assess whether the performance of IGRA test would be affected by the manner of presentation to the hospital. The emergency departments (ED) of Korean hospital often receive patients with pulmonary infiltrates associated with acute derangement. Since Korea is an area with an intermediate burden of active tuberculosis [11], it is frequently challenging for clinicians to distinguish between TB and non-TB. In such clinical situation, IGRA test for the diagnosis of TB infection may be helpful as a supplemental tool for the diagnostic exclusion of active TB [12]. Thus, we investigated the performance of IGRA test in these ED inpatients and compared it with its performance in outpatients with pulmonary TB. In addition, we sought to identify the factors that influence the performance of IGRA test in both inpatients and outpatients with active pulmonary TB.

## Methods

### Study population and design

At the Kyungpook National University Hospital between March 1, 2009 and June 30, 2010, patients older than 18 years who presented at the ED with acute respiratory symptoms, and whose chest radiographs exhibited pulmonary infiltrates were hospitalized and routinely tested with one of two QuantiFERON tests (QFT), namely, QuantiFERON^®^-TB Gold (QFT-G) and QuantiFERON^®^-TB Gold In-Tube (QFT-GIT) (both are from Cellestis Ltd., Carnegie, Australia). The patients who received a QFT test at the ED were prospectively enrolled as an inpatient group of this study. Patients with HIV infection, known lung cancer, or overt lung mass suggesting lung cancer were excluded. Moreover, the patients who were diagnosed with pulmonary TB and received a QFT test before the initiation of anti-TB treatment at the outpatient department (OPD) during the same period were recruited as a control group. The patients in the control group were not hospitalized until the end of treatment except if problems arose, such as non-respiratory disease or drug intolerance. The clinical, laboratory, and radiographic data and the results of QFT test were collected in both groups.

The diagnosis of TB was definitely confirmed for all TB patients by culturing *Mycobacterium tuberculosis *in their sputum or bronchial aspirate. Diseases other than TB were finally diagnosed by pathology, culture results, and follow-up chest radiographs. In patients with pulmonary TB, the radiographic extent of the lesion was simply classified as mild, moderate, and advanced groups. Thus, lesions extending to within one-third of the unilateral lung field, within the unilateral lung field beyond one-third of the field, and beyond the unilateral lung field were classified as mild, moderate, and advanced, respectively [10]. The study was approved by the institutional research board of Kyungpook National University Hospital, and informed consent was waived.

### QFT test (QFT-G/QFT-GIT)

The QFT-G test was replaced by the QFT-GIT test during the study period. In total, 128 and 249 patients were tested with QFT-G and QFT-GIT, respectively. The tests were performed according to the recommendations of the manufacturers [13] by trained technicians. In brief, the tests consisted of a negative control, a positive control, and two sample wells (whole-blood stimulated with either early secretary antigenic target 6 (ESAT-6) or culture filtrate protein (CFP-10)) for the QFT-G test or three heparinized tubes (a test tube containing the ESAT-6, CFP-10, and TB7.7 peptide cocktail, a positive control, a negative control) for the QFT-GIT test. Whole-blood specimens contained in wells or tubes were incubated for 16-20 h at 37°C in a carbon dioxide incubator. The IFN-γ titer of the negative well is considered to be the background value and is subtracted from the values of the mitogen control and the antigen-stimulated wells. The samples are considered to be positive if their TB antigen-stimulated IFN-γ titers are ≥0.35 IU/ml, regardless of the positive control result. The samples are considered to be negative if their TB antigen-stimulated IFN-γ titers are <0.35 IU/ml and their positive control titers are ≥0.5 IU/ml. The test result is considered to be indeterminate if TB antigen- and mitogen-stimulated IFN-γ titers are <0.35 IU/ml and <0.5 IU/ml, respectively [13].

### Statistical analysis

The χ^2 ^or the Fisher exact test, if there were ≤ 5 observations, was used to compare the findings between the two groups. The T-test was performed to determine the statistical significance of differences between means of some variables. The effects of age, sex, and immune status on the probability of obtaining an indeterminate QFT result were analyzed by logistic regression analysis. *P *values < 0.05 were considered significant. Statistical analyses were performed by using statistical software (SPSS for Windows, version 17.0).

## Results

### Baseline characteristics of the study population

Overall, 284 inpatients and 93 outpatients were recruited. Baseline characteristics of all 377 patients are shown in Table 1. Among inpatients, 88 patients (31.0%) were diagnosed with pulmonary TB. The remaining inpatients were finally diagnosed with pneumonia (n = 178), organizing pneumonia (n = 7), nontuberculous mycobacterial lung disease (n = 5), aspergillosis (n = 4), and lung cancer (n = 2), respectively. Twenty-six patients had risk factors for immunosuppression, such as renal failure (n = 11), malignancies that were not receiving anti-cancer treatments at the time of testing with QFT (n = 7), and immunosuppressive therapies including cancer chemotherapy (n = 9) and systemic steroids (n = 2).

**Table 1:** Baseline characteristics of the study population

Characteristics	Inpatients			Outpatients
		
	Total (n = 284 )	* Non- TB (n = 196)	TB (n = 88)	TB (n = 93)
Age, yr	63 ± 16.5	65 ± 14.5	59 ± 19.8	52 ± 19.5
Male	206 (72.5)	150 (76.5)	56 (63.6)	50 (53.8)
Body mass index	21.4 ± 3.50	21.7 ± 3.42	20.8 ± 3.61	21.7 ± 3.08
Prior TB history	57 (20.1)	39 (19.9)	18 (20.5)	16 (17.2)
Underlying conditions				
Renal failure	11 (3.9)	9 (4.6)	2 (2.3)	0 (0)
Malignancy†	4 (1.4)	2 (1.0)	2 (2.3)	3 (3.2)
Immunosuppressive therapy‡	11 (3.9)	7 (3.6)	4 (4.5)	0 (0)

Data are shown as mean ± SD or No. (%). TB, tuberculosis.

* Patients with pneumonia (n = 178), organizing pneumonia (n = 7), nontuberculous mycobacterial lung disease (n = 5), aspergillosis (n = 4), and lung cancer (n = 2).

† Patients with a diagnosis of cancer who were not receiving anti-cancer treatment, including lung cancer (n = 2), hypopharyngeal cancer (n = 1), and gastric cancer (n = 1) for inpatients; hepatocellular carcinoma (n = 1), glottic cancer (n = 1), and gastric cancer (n = 1) for outpatients.

‡ Patients receiving cancer chemotherapy (n = 9 ) or systemic steroids (>10 mg/day of prednisone for ≥ 1 month) (n = 2).

### Performance of QFT test in inpatients with pulmonary infiltrates and outpatients with TB

Table 2 summarizes the QFT results of the 377 patients divided according to whether they were inpatients or outpatients; the inpatients were also further divided according to whether non-TB or TB was diagnosed. Of the 284 inpatients, 84 (29.6%) had an indeterminate QFT result. All of them were attributed to the absence of sufficient response to mitogen control. Of the inpatients with non-TB and TB, 35.2% and 17.0% had indeterminate QFT results, respectively. This difference was statistically significant (p = 0.002). Of the 93 outpatients, all of whom had pulmonary TB, there was only one indeterminate QFT result (1.1%). The inpatients with pulmonary TB were significantly more likely to have indeterminate QFT results than the outpatients (17.0% vs. 1.1%, respectively, p < 0.001). In addition, the sensitivity of QFT test for the diagnosis of active TB was significantly lower for inpatients than outpatients (58.0% vs. 82.8%, p < 0.001). The negative predictive value of QFT test was 82.1% in the inpatient group.

**Table 2:** Performance of QuantiFERON test in inpatients with pulmonary infiltrates and outpatients with pulmonary tuberculosis

QuantiFERON†	Inpatients			*p value*	Outpatients	*p value*
				
	Total (n = 284)	* Non-TB (n = 196)	TB (n = 88)		TB (n = 93)	
Indeterminate	84 (29.6)	69 (35.2)	15 (17.0)	0.002^*a*^	1 (1.1)	<0.001^*b*^
Determinate	200 (70.4)	127 (64.8)	73 (83.0)		92 (98.9)	<0.001^*c*^
Positive	77 (27.1)	26 (13.3)	51 (58.0)		77 (82.8)	
Negative	123 (43.3)	101 (51.5)	22 (25.0)		15 (16.1)	

Data are shown as mean ± SD or No. (%). TB, tuberculosis.

^† ^QuantiFERON test was performed by using QuantiFERON^®^- TB Gold or QuantiFERON ^®^-TB Gold In-Tube.

* Patients with pneumonia (n = 178), organizing pneumonia (n = 7), nontuberculous mycobacterial lung disease (n = 5), aspergillosis (n = 4), and lung cancer (n = 2).

^*a *^Comparison of proportion of indeterminate vs. determinate results between in patients with non-TB and TB.

^*b *^Comparison of proportion of indeterminate vs. determinate results between inpatients and out patients with TB.

^*c *^Comparison of positive QuantiFERON test results between inpatients and outpatients with TB.

### Univariate and multivariate analysis of factors associated with indeterminate QFT results in inpatients with pulmonary infiltrates

The clinical and laboratory findings of the 84 inpatients whose QFT results were indeterminate were compared to those of the remaining 200 inpatients, whose QFT results were determinate. Univariate analysis revealed that patients with indeterminate QFT results were significantly older, more likely to have lymphocytopenia (lymphocyte count less than 1000 cells/μL), and had lower lymphocyte counts, lower albumin concentrations, higher C-reactive protein (CRP) levels than those with determinate results (Table 3). Gender, body mass index, prior TB treatment history, and underlying conditions including immunosuppressive therapies did not associate significantly with the occurrence of indeterminate results in inpatients. Multivariate analysis then revealed that patients with indeterminate QFT results had significantly lower albumin concentrations and higher CRP levels than those with determinate results. They were also significantly more likely to have lymphocytopenia.

**Table 3:** Univariate and multivariate analysis of factors associated with indeterminate QuantiFERON results in inpatients with pulmonary infiltrates

Covariate	Determinate (n = 200)	indeterminate (n = 84)	Univariate		Multivariate	
				
			Odds ratio	95% confidence interval	Odds ratio	95% confidence interval
Age, yr	62 ± 17.1	67 ± 14.5	1.018	1.001-1.035		
Lymphocytes cells/μL	1170 ± 935	764 ± 451	0.999	0.998-0.999		
Lymphocytopenia*						
Absence	99 (49.5)	19 (22.6)	1.0		1.0	
Presence	101 (50.5)	65 (77.4)	3.353	1.875-5.998	2.688	1.424-5.075
Albumin, g/dL	3.4 ± 0.60	3.0 ± 0.54	0.307	0.190-0.495	0.412	0.245-0.694
ESR, mm/hr	58.3 ± 35.88	67.2 ± 32.52	1.007	1.000-1.015		
CRP, mg/dL	9.2 ± 8.43	15.7 ± 9.65	1.078	1.046-1.110	1.060	1.027-1.094

Data are shown as mean ± SD or No. (%).

ESR, erythrocyte sedimentation rate; CRP, C-reactive protein.

QuantiFERON test was performed by using QuantiFERON^®^- TB Gold or QuantiFERON ^®^- TB Gold In-Tube.

* Lymphocytopenia; < 1,000 cells/μL.

### Comparison of inpatients and outpatients with pulmonary TB with regard to the factors associated with non-positive QFT results

Since the inpatients and outpatients with pulmonary TB differed significantly with regard to the sensitivity of the QFT, we sought to identify the factors that were associated with non-positive QFT results in the two groups. Table 4 shows the laboratory and radiographic findings of both the inpatients with TB and the outpatients after they had been divided according to whether their QFT result was non-positive. Baseline characteristics were not significantly different between patients with positive and non-positive QFT results in both subgroups (data not shown). The inpatients with non-positive QFT results had significantly lower mean lymphocyte counts, lower mean albumin concentrations, and a higher rate of lymphocytopenia than those with positive QFT results. In contrast, the outpatients with non-positive QFT results had significantly higher mean erythrocyte sedimentation rates (ESR) and more severe disease (as observed by radiography) than the outpatients with positive QFT results. Thus, the inpatients differed from the outpatients in terms of the clinically presented factors that were associated with a non-positive QFT result.

**Table 4:** Comparison of inpatients and outpatients with pulmonary tuberculosis with regard to the factors associated with non-positive QuantiFERON results

Covariates	Inpatients		*P value*	Outpatients		*P value*
				
	Positive QFT (n = 51)	Non-positive QFT (n = 37)		Positive QFT (n = 77)	Non-positive QFT (n 16)	
Laboratory findings						
WBC, cells/μL	8566 ± 3438	10604 ± 5464	0.050	7723 ± 2372	7526 ± 2212	0.761
Lymphocytes, cells/μL	1203 ± 509	885 ± 440	0.003	1 787 ± 642	1508 ± 504	0.105
Lymphocytopenia*	20 (39.2)	24 (64.9)	0.018	7 (9.3)	1 (7.7)	1.0
Albumin, g/dL	3.6 ±0.64	3.1 ± 0.59	0.003	4.4 ± 0.40	4.1 ± 0.56	0.051
ESR, mm/hr	60.5 ± 35.15	62. 5 ± 35.97	0.806	33.9 ± 27.1	58.1 ± 33.4	0.004
CRP, mg/dL	6.5 ± 6.21	8.3 ± 6.83	0.230	2.1 ± 2.92	0.44 ± 0.30	0.452
Radiographic extent of disease						
Mild	19 (37.3 )	13 (35.1 )	0.589	55 (71.4 )	6 (37.5)	0.020
Moderate	19 (37.3)	11 (29.7 )		21 (27.3)	10 (62.5)	
Advanced	13 (25.5)	13 (35.1)		1 (1.3)	0	

Data are shown as mean ± SD or No. (%).

WBC, white blood cells; ESR, erythrocyte sedimentation rate; CRP, C-reactive protein; QFT = QuantiFERON (performed by QuantiFERON^®^-TB Gold or QuantiFERON^®^-TB Gold In-Tube).

* Lymphocytopenia; < 1,000 cells/μL.

## Discussion

The main findings of this study were: 1) Inpatients admitted to the ED with either pulmonary TB or non-TB diseases were significantly more likely to have indeterminate QFT results than outpatients with pulmonary TB; 2) The sensitivity of QFT test for the diagnosis of pulmonary TB showed significant difference between inpatients (58.0%) and outpatients (82.8%); 3) Independent factors that were associated with indeterminate QFT results in inpatients were lymphocytopenia, lower albumin concentrations, and higher CRP levels; 4) The inpatients with TB differed from the outpatients in terms of the risk factors that were associated with non-positive QFT results.

Previous large studies have demonstrated that the QFT, when used routinely in hospitals to diagnose TB infection, often yields indeterminate results [6,7,9,10]. However, these studies only analyzed cohorts with a high proportion of immunosuppressed individuals [6,7], children [9], or preselected patients with suspected TB and latent TB infection [10]. In addition, the proportion of patients with active TB in these studies was small. Moreover, these studies did not discriminate between inpatients and outpatients. Therefore, the results of these studies may not adequately reflect the performance of the QFT in patients who present at an ED with pulmonary infiltrates. Our study demonstrates the performance of the QFT in these inpatients. Our results suggest that the performance of the QFT in an ED can vary depending on the disease that is involved and may be significantly worse than its performance in an OPD setting. This is the first report of the performance of the QFT when it is applied routinely in an ED to differentially diagnose patients who present with acute respiratory symptoms and pulmonary infiltrates.

The QFT sensitivity of all of the TB patients, including inpatients and outpatients, was 70.7%, which is within the range of QFT sensitivities of prior reports, including those obtained by meta-analysis [14]. Among prior reports, Mazurek et al. [15] reported QFT-G sensitivity of 69.6% in patients with culture-confirmed pulmonary TB, which is very similar to the sensitivity in the same population in our study. However, our data showed a significant difference of QFT sensitivity between inpatients and outpatients with culture-confirmed pulmonary TB. In general, inpatients with TB admitted to the ED are expected to have a more severe form of TB than outpatients with TB. Thus, our data suggests that the sensitivity of the QFT test may be significantly different depending on disease severity in the study population.

Previous studies have shown that host factors that are significantly associated with indeterminate QFT results are immunosuppressive therapies, extremes of age, lymphocytopenia, hypoalbuminemia, and HIV-infection with lower CD4 lymphocyte counts [6,7,9,10,16]. Other causes of indeterminate QFT results include pre-analytical errors such as the storage of tubes outside the recommended temperature range, the over- or under-filling of tubes with blood, insufficient mixing, and incomplete washing of the enzyme-linked immunosorbent assay plate [13]. However, while recent technical modifications of the QFT, such as vortexing or immediate incubation, have reduced the rate of indeterminate QFT-GIT results [17,18], they are unlikely to be able to overcome the effect of host factors that lead to low cell-mediated immune responses. Indeed, the high rate of indeterminate results observed in our ED setting did not seem due to errors in the testing process, as the manufacturer's instructions were followed strictly and the rate of indeterminate QFT results in outpatients with pulmonary TB (whose samples were handled in an identical manner to those of inpatients) was very low. Therefore, we believe that patient-specific factors rather than technical problems are the main reasons for the high rate of indeterminate QFT results in our inpatients. Further analysis revealed that those factors are lymphocytopenia, hypoalbuminemia, and increased CRP levels. This list differs somewhat from the list of host factors mentioned above that were identified by previous studies that were based on routine hospital use [6,7,10].

The association of lymphocytopenia with indeterminate QFT results is not surprising as it is well known that lymphocytopenia can decrease the production of IFN-γ. Moreover, as suggested by a previous study [10], the association of indeterminate QFT results with hypoalbuminemia may reflect a poor nutritional status that compromises the immune system. However, our study is the first to show an association between higher CRP levels and indeterminate QFT results. Since CRP is a major positive acute phase reactant [19], higher CRP levels are likely to reflect more intensive inflammation, which in turn suggests that patients with indeterminate QFT results have higher levels of inflammation than those with determinate QFT results. Notably, since albumin is a major negative acute phase reactant [19], a higher inflammatory status result may explain the association between hypoalbuminemia and poor QFT results better than the malnutrition hypothesis. Moreover, lymphocytopenia is an extremely common feature of the acute response to stress in acutely ill patients, regardless of the nature of the underlying illness [20]. Although this may reflect the small numbers of patients with immunosuppressive therapies or old age in our study, it is also possible that these underlying conditions play less important roles in acute status than in chronic status. In addition, since the effects of underlying conditions, which can vary between individuals and during the clinical course, would be ultimately reflected in lymphocyte counts and acute phase reactants, these parameters may be good objective markers that indicate the strength of cellular immunity and the risk of poor QFT performance.

Hospitalization-related stress is known to promote the systemic secretion of glucocorticoids and catecholamines, which, in turn, influence immune responses [21]. In particular, glucocorticoids are known to suppress the production of TNF-α, IFN-γ, and IL-2 in animals and humans both *in vitro *and *in vivo *[22,23]. In addition, catecholamines inhibit the development of Th1-type cells, thereby inhibiting IFN-γ production [24]. Thus, the fact that inpatients with pulmonary TB have higher rates of indeterminate QFT result than outpatients may partly reflect the derangement of immune responses caused by hospitalization at the ED.

Lymphocytopenia and hypoalbuminemia were found to be risk factors of non-positive QFT results in inpatients with active TB. This is consistent with our observation that clinically significant inflammation associated with indeterminate QFT results in inpatients in general. However, acute systemic inflammation did not seem to be strongly associated with non-positive QFT results in OPD-based TB patients. Indeed, the outpatients with non-positive QFT results did not differ significantly from the other outpatients in terms of lymphocyte counts and the levels of major acute phase reactants such as albumin and CRP. However, the outpatients with non-positive QFT results did have significantly higher ESR values than the other outpatients. Since CRP concentrations change rapidly whereas the ESR changes relatively slowly [19], it may be that the OPD-based TB patients with non-positive QFT results were at a more chronic and stable stage of the inflammatory process (namely, a stage where CRP responses were no longer being elicited).

Radiographically more severe disease was also significantly associated with non-positive QFT results in the outpatients. This is consistent with the suggestion that the limited sensitivity of IGRAs in patients with active TB may relate to the immunological incapacity of the host to contain mycobacterial replication [25]. Moreover, IFN-γ secretion has been shown to correlate inversely with disease severity in patients with TB [25-27]. These observations may explain why outpatients with non-positive QFT results had extensive disease more frequently than the other outpatients. In a certain aspect, radiographically more extensive diseases may be in line with higher ESR values shown in TB outpatients with non-positive QFT results.

Taken together, these observations suggest that the level of immune competence varies among patients with mycobacterial diseases. The different clinical presentations of TB appear to be associated with the degree of alteration of the immune response against the mycobacteria. T cell dysfunction by alterations in the expression of several T cell signal-transduction proteins was reported in patients with pulmonary TB [28]. In addition, severe TB in a macaque model induces unbalanced up-regulation of immune gene networks, causing overexpression of genes encoding inflammatory cytokines and chemokines without proportional increases in antigen-specific cellular responses [29]. Thus, TB inpatients, who are expected to have more severe disease than OPD-based TB patients, are likely to have more intense inflammation and more severe T cell dysfunction, perhaps explaining why more intense inflammation is associated with indeterminate result of the QFT test.

The main limitation of the present study is the fact that the study population was tested by two different QFTs (QFT-G and QFT-GIT) because the QFT-G was replaced by the newer generation test QFT-GIT during the study period. However, a recent meta-analysis that also included the indeterminate result rates has found that QFT-G and QFT-GIT may be similarly sensitive [14]. Thus, this factor is not likely to have significantly affected our results. Another potential limitation is the fact that only a small number of patients received immunosuppressive treatment. Thus, the power of our study to detect the impact of immunosuppressive therapy on QFT performance was probably rather weak. In addition, the numbers of inpatients with TB and outpatients after they were subgrouped according to their QFT results were small. As well, our data could not directly determine the mechanism by which more intense inflammation, as evidenced by higher CRP levels and lower albumin concentrations, is associated with indeterminate results in the QFT test. These relationships will require further study. Finally, our observations cannot be generalized to all populations with pulmonary infiltrates because most of non-TB population were consisted of patients with pneumonia (91%).

## Conclusion

Our findings indicate that, unlike QFT of outpatients with pulmonary TB, QFT performed in the ED for inpatients with pulmonary infiltrate frequently return indeterminate results. In addition, our study showed that inpatients and outpatients with pulmonary TB may differ in terms of the risk factors that are associated with non-positive QFT results.

## Competing interests

The authors declare that they have no competing interests.

## Authors' contributions

YJL, JL, and CHK were responsible for planning the study, analyzing the results and writing the manuscript. YYK, SIC, JYP and THJ participated in the acquisition of data. DIW carried out monitoring of QFT testing. All authors have read and approved the manuscript.

## Pre-publication history

The pre-publication history for this paper can be accessed here:

http://www.biomedcentral.com/1471-2334/11/107/prepub
